# Pharmacokinetic and pharmacodynamic analysis of fulvestrant in preclinical models of breast cancer to assess the importance of its estrogen receptor-α degrader activity in antitumor efficacy

**DOI:** 10.1007/s10549-019-05454-y

**Published:** 2019-09-27

**Authors:** Suzanne E. Wardell, Alexander P. Yllanes, Christina A. Chao, Yeeun Bae, Kaitlyn J. Andreano, Taylor K. Desautels, Kendall A. Heetderks, Jeremy T. Blitzer, John D. Norris, Donald P. McDonnell

**Affiliations:** 1 Department of Pharmacology and Cancer Biology, Duke University School of Medicine, Box 3813, Durham, NC 27710 USA; 2 Potrero Hill Therapeutics, San Francisco, CA 94110 USA

**Keywords:** Selective estrogen receptor downregulator, Fulvestrant, Endocrine resistant breast cancer

## Abstract

**Purpose:**

Fulvestrant is a selective estrogen receptor downregulator (SERD) that is approved for first- or second-line use as a single agent or in combination with cyclin dependent kinase or phosphatidylinositol 3-kinase inhibitors for the treatment of metastatic breast cancer. Fulvestrant exhibits exceptionally effective antitumor activity in preclinical models of breast cancer, a success that has been attributed to its robust SERD activity despite modest receptor downregulation in patient tumors. By modeling human exposures in animal models we probe the absolute need for SERD activity.

**Methods:**

Three xenograft models of endocrine therapy-resistant breast cancer were used to evaluate the efficacy of fulvestrant administered in doses historically used in preclinical studies in the field or by using a dose regimen intended to model clinical exposure levels. Pharmacokinetic and pharmacodynamic analyses were conducted to evaluate plasma exposure and intratumoral ER downregulation.

**Results:**

A clinically relevant 25 mg/kg dose of fulvestrant exhibited antitumor efficacy comparable to the historically used 200 mg/kg dose, but at this lower dose it did not result in robust ER downregulation. Further, the antitumor efficacy of the lower dose of fulvestrant was comparable to that observed for other oral SERDs currently in development.

**Conclusion:**

The use of clinically unachievable exposure levels of fulvestrant as a benchmark in preclinical development of SERDs may negatively impact the selection of those molecules that are advanced for clinical development. Further, these studies suggest that antagonist efficacy, as opposed to SERD activity, is likely to be the primary driver of clinical response.

**Electronic supplementary material:**

The online version of this article (10.1007/s10549-019-05454-y) contains supplementary material, which is available to authorized users.

## Background

Estrogens play a critical role in the development of female reproductive tissues and contributes to the development and progression of breast cancer. Patients that present with estrogen receptor α (ER)-positive disease are generally treated with an antiendocrine therapy in the adjuvant setting, consisting of either the selective estrogen receptor modulator (SERM) tamoxifen or an aromatase inhibitor (AI, e.g., anastrozole, letrozole, exemestane) [1]. These adjuvant therapies have proven effective to reduce the incidence of progression to metastatic disease. Patients that present with or progress to advanced metastatic breast cancer are also typically treated with an antiendocrine therapy as part of a more aggressive treatment regimen [2]. In this setting, these treatments are not curative, with a significant number of cancers exhibiting de novo or rapidly acquired resistance to existing antiestrogens and aromatase inhibitors. After progression during SERM and AI therapy, other endocrine therapies can be effective, including the steroidal selective estrogen receptor degrader (SERD) fulvestrant, megestrol acetate (a progesterone receptor agonist), or high-dose estrogens [3–5].

SERDs are a particularly intriguing option because they have the dual action of both eliminating ER expression and directly inhibiting activity. The first drug of the SERD class to be developed, fulvestrant (faslodex), demonstrated efficacy in preclinical in vitro and in vivo models of breast cancer either sensitive or resistant to tamoxifen (standard of care breast cancer treatment at that time) and entered clinical trials with the expectation of similar efficacy in the treatment of relapsed/progressing breast tumors [6–8]. Despite ongoing efforts by several academic and commercial entities to develop an oral SERD, fulvestrant remains the only drug in this class approved for the treatment of breast cancer.

Since its initial preclinical evaluation in xenograft tumor models in mice [7], fulvestrant is generally administered as a weekly injection of 5 mg/mouse (~ 200 mg/kg, depending on strain). However, the body surface area (BSA) based inter-species conversion calculation embraced by the US Food and Drug Administration indicates that a mouse dose equivalent to that used clinically would be 100 mg/kg per four-week cycle (25 mg/kg/week) [9]. While an argument can be made that the more rapid turnover of mouse serum albumin (3 days vs. 3 weeks in humans) might require a higher exposure, there remains a clear possibility that the dosing regimen widely used preclinically in mouse studies likely exceeds that approved for clinical use by 8-fold, overestimating its potential activity and inappropriately setting the benchmark for other therapies. Therefore, we sought to conduct a thorough PK/PD analysis of fulvestrant exposure in mice and relate this to efficacy in relevant breast cancer tumor models. We further probed the relationship between drug exposure, antitumor efficacy, and receptor turnover, an exceptionally important unanswered question in this field.

## Materials and methods

### Reagents and source

Tamoxifen treatment pellets were purchased from Innovative Research of America. Fulvestrant, AZD9496, GDC0810, and bazedoxifene were purchased from MedChemExpress. Estradiol was purchased from Sigma-Aldrich, Inc. Antibodies used for immunoblot detection included the following: estrogen receptor (ER)—HC-20 or H184, Santa Cruz Biotechnology; actin—A5441, Sigma-Aldrich, Inc.; vinculin—13901S, Cell Signaling.

### Xenograft tumor studies

All procedures were approved by the Duke University Institutional Animal Care and Use Committee (IACUC) prior to initiating the experiment.

#### TamR study 1

TamR tumors were implanted orthotopically (8 mm^3^ fragment inserted sc into the mammary fat pad) into 65 ovariectomized tamoxifen-treated (5 mg/60 days pellet, Innovative Research of America) female *Nu/J* mice (~ 6 weeks of age). Tumor volume and body weight were measured 3X weekly until tumors reached ~ 0.1–0.15 cm^3^ volume (*l* × *w*^2^ × 0.5). Mice were then randomized (*n* = 8–9) to 4 weekly treatments with 0, 25, 50, 100, or 200 mg/kg fulvestrant injected sc (5% DMSO/95% corn oil) with continued tumor measurement and weight monitoring. After 28 days of treatment, animals were euthanized by CO_2_ exposure, followed immediately by cardiac puncture for blood collection. Plasma and tumor tissues were cryopreserved for future analysis.

#### TamR study 2

50 female *Nu/J* mice were ovariectomized and received Tam treatment pellet, tumor implantation, and monitoring as described above. When tumors reached ~ 0.1–0.15 cm^3^ volume, mice were randomized (*n* = 8) to daily po treatment with vehicle (9/0.5/0.5/90 PEG 400/PVP/Tween 80/0.5% CMC), AZD9496 (10 mg/kg), GDC0810 (25 mg/kg), or bazedoxifene (12.5 mg/kg). After 28 days of treatment, animals were euthanized and tissues harvested as above.

#### LTED study

65 female *Nu/J* mice were ovariectomized and 1 week later LTED tumor fragments were implanted orthotopically as described above and similarly monitored. Mice were then randomized (*n* = 8–9) to daily po treatment with vehicle (9/0.5/0.5/90 PEG 400/PVP/Tween 80/0.5% CMC), AZD9496 (10 mg/kg), GDC0810 (25 mg/kg), or bazedoxifene (12.5 mg/kg). Fulvestrant-treated mice (25 mg/kg sc weekly) also received daily treatment with vehicle po. After 28 days of treatment, animals were euthanized and tissues harvested as above.

#### HCC1428 study

40 female *Nu/J* mice were ovariectomized with concurrent initiation of estradiol treatment (0.75 µg/ml in drinking water). Fragments of an HCC1428 xenograft tumor were implanted as described above. When tumors reached ~ 0.1–0.15 cm^3^ volume, animals were randomized to weekly injection of vehicle or 25 mg/kg fulvestrant (as above).

### Immunoblot analysis of tumor tissue

Frozen tissues were pulverized under LN_2_ prior to protein extraction of powdered tissues using RIPA buffer (50 mM Tris, pH 8, 150 mM NaCl, 1% NP-40, 0.5% deoxycholate, 0.02% SDS, 1 mM EDTA). 25 µg of cleared extracts were resolved by SDS-PAGE prior to transfer to PVDF membrane and immunoblot analysis by standard methods. Bands detected were quantitated using ImageJ per standard methods [10].

### Real-time quantitative PCR analysis of tumor tissue

Total RNA was extracted from pulverized frozen tissues using the Aurum total RNA extraction kit (Bio-Rad) as per kit instructions. Following cDNA synthesis (iScript, Bio-Rad), RT-qPCR analysis of cDNA samples was performed using iQ SYBR Green Supermix (Bio-Rad). mRNA abundance was calculated using the ΔΔC_T_ method [11].

### Statistical analyses

Tumor growth data were subjected to exponential growth curve analysis constrained to share an initial value, and to 2-way ANOVA analysis followed by Bonferroni multiple comparison test. Significant differences as compared to the vehicle treated control (*p* < 0.05) were detected for multiple groups at several time points (indicated on graphs). Groups showed equivalent variance (10–15% with normal distribution) throughout all time points, justifying the statistical analyses that were selected. ER expression levels were evaluated using 1-way ANOVA analysis followed by Bonferroni multiple comparison test. Percent tumor growth inhibition (TGI) at each day of measurement was calculated using the equation %TGI = [1 − (mean tumor volume of treated)/(mean tumor volume of vehicle)].

## Results

### Fulvestrant exhibits antitumor efficacy in breast cancer tumors at a clinically relevant dose

**Fig. 1:**
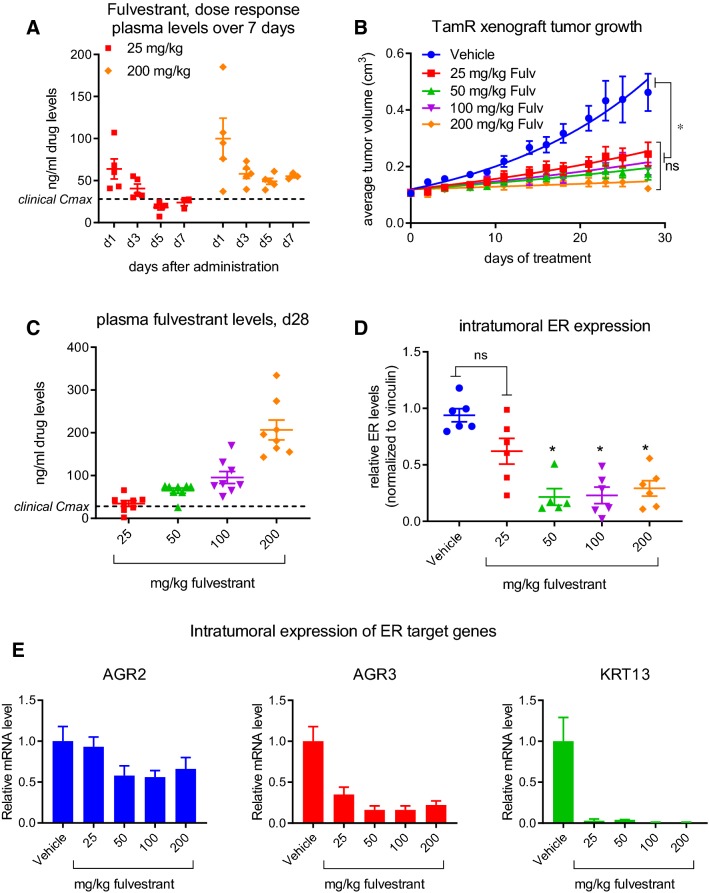
Evaluation of PK/PD and efficacy of fulvestrant in an endocrine therapy-resistant tumor model. **a** Circulating plasma levels of fulvestrant in *Nu:J* mice 1, 3, 5, or 7 days following a single administration of 25 or 200 mg/kg fulvestrant. Detection of fulvestrant in mouse plasma was conducted via LC/MS/MS (Confluence Discovery Technologies, Saint Louis, MO). **b** Tamoxifen-treated *Nu:J* mice bearing TamR (tamoxifen-resistant) xenograft tumors were randomized to treatment with vehicle or fulvestrant (25–200 mg/kg). Data presented indicate the average tumor volume for each group (mean ± SEM) at each time point. *Significant (*p* > 0.05) inhibition of tumor growth was observed for all doses of fulvestrant (2-way ANOVA analysis followed by Bonferroni multiple comparison test). **c** Levels of fulvestrant were evaluated in plasma retained from mice included in (**b**). **d** ER levels present in tumors harvested from mice included in **b** were analyzed by western blotting of tissue extracts. A significant decrease in ER levels was observed for 50, 100, and 200 mg/kg doses *p < 0.05 (1-way ANOVA analysis followed by Bonferroni multiple comparison test). Primary western blot images are included in Suppl. Fig. 1. **e** The expression of tamoxifen responsive ER target genes AGR2, AGR3, and KRT13 in cDNA generated from tumors harvested in **b** were analyzed by RT-qPCR

As a first step to evaluate in vivo exposure of fulvestrant in mice, we initially conducted a simple PK analysis in which *Nu:J* mice received a single injection of 25 or 200 mg/kg fulvestrant, and plasma was collected for analysis from mice euthanized 1, 3, 5, or 7 days after administration. The circulating levels of fulvestrant in plasma samples retained were evaluated by LC/MS/MS analysis. Both doses resulted in highest levels detected 1 day after administration (Fig. 1a), but the ensuing plateau observed for the 25 mg/kg dose best approximated the approximate 28 ng/ml C_max_ observed per current protocol in patients [12].

In order to assess the relationship between dose and efficacy of fulvestrant on the growth of a clinically relevant xenograft model of endocrine therapy-resistant breast cancer, tamoxifen-resistant (TamR) xenograft tumors were established orthotopically in ovariectomized tamoxifen-treated (5 mg/60 days administered by continuous release pellet) female *Nu:J* mice. When tumors measured 0.1–0.15 cm^3^ volume, mice were randomized to 4 weekly injections with Vehicle or fulvestrant (25, 50, 100, or 200 mg/kg). A significant inhibition of tumor growth was observed for all doses of fulvestrant as compared to the vehicle control (Fig. 1b), and no significant differences could be detected between doses.

Circulating plasma levels of fulvestrant present at euthanasia (7 days after final dose) were evaluated as above (Fig. 1c). A linear relationship between dose and plasma levels was noted, with mean values of 34.6, 64.9, 95.6, and 207 ng/ml (57, 107, 157, and 340 nM) being detected for 25, 50, 100, and 200 mg/kg doses, respectively. It is worth noting that after 4 weekly administrations, the circulating plasma levels observed for the 25 mg/kg dose best approximated the clinical C_max_, as would be expected using the BSA calculation.

The efficient tumor growth inhibition observed for fulvestrant at doses several fold lower than the 200 mg/kg dose historically used preclinically led us to evaluate the pharmacodynamic relationship between the dose administered and ER downregulation, as the clinical efficacy of fulvestrant has been attributed at least in part to its ability to reduce ER expression. A dose dependent downregulation of ER expression was observed in the harvested tumor tissues when evaluated by immunoblot of the cleared lysates derived from fulvestrant-treated tumors (relative ER levels are presented in Fig. 1d, primary blots are included in Online Resource 1a). Interestingly, for those tumors treated with the 25 mg/kg dose, a wide range of ER levels were detected, which is again reminiscent of the clinical observations with fulvestrant, as the extent of ER downregulation observed comparing pre- and post-treatment biopsies can vary widely [13]. Significant (*p* < 0.001) downregulation of ER was observed only for 50, 100, and 200 mg/kg doses despite equivalent inhibition of tumor growth by all doses administered. These data question the importance of SERD activity to the antitumor efficacy of fulvestrant.

To evaluate further the relationship between ER downregulation and inhibition of ER activity, the mRNA expression of ER target genes known to be responsive to tamoxifen in this tumor model (Online Resource 1b) was evaluated by real-time quantitative PCR (RT-qPCR). As shown in Fig. 1e, all of the doses of fulvestrant administered resulted in dramatic downregulation of the expression of anterior gradient protein 3 (AGR3) and keratin 13 (KRT13). Interestingly, a modest reduction in AGR2 expression was observed only for those doses of fulvestrant that resulted in quantitative downregulation of ER protein. This latter result is consistent with previous findings from our laboratory demonstrating an enhancer switch in the *AGR2* gene in this tamoxifen-resistant tumor model, thereby reducing the dependence of *AGR2* expression on ER activity [14].

### When administered at a clinically relevant dose, SERDs exhibit similar efficacy and pharmacodynamics

**Fig. 2:**
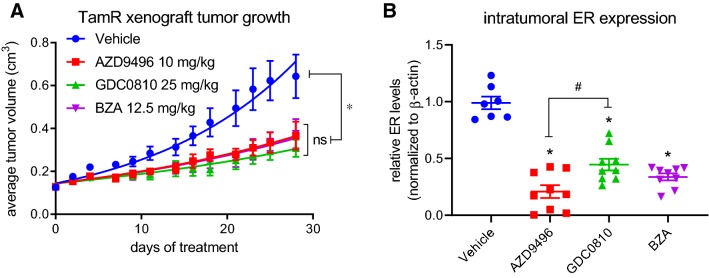
Clinically relevant oral SERDs exhibit similar efficacy in tumor growth inhibition. **a** Tamoxifen-treated *Nu:J* mice bearing TamR xenograft tumors were randomized to treatment with vehicle (po qd), AZD9496 (10 mg/kg po qd), GDC0810 (25 mg/kg po qd), or BZA (12.5 mg/kg po qd). Data presented indicate the average tumor volume for each group (mean ± SEM) at each time point. *As compared to the vehicle control, significant (*p* > 0.05) inhibition of tumor growth was observed for all treatments, while no significant differences were noted between treatments (2-way ANOVA analysis followed by Bonferroni multiple comparison test). **b** ER levels present in tumors harvested from mice included in **a** were analyzed by western blotting of tissue extracts. *A significant decrease in ER levels was observed for all SERD treatments (1-way ANOVA analysis followed by Bonferroni multiple comparison test). ^#^*p* < 0.05: Significantly less ER turnover was observed following GDC0810 treatment as compared to AZD9496. Primary western blot images are included in Suppl. Fig. 2

We and others have previously conducted in vivo studies comparing the efficacy of oral SERDs and fulvestrant [15–17]. One similarity between these studies is the greater effectiveness generally observed for fulvestrant (200 mg/kg in mice) in endocrine therapy-resistant breast tumor models without ER mutations as compared to more recently developed SERDs administered at a BSA-calculated clinically relevant dose. Upon observing the comparable efficacy of fulvestrant above over a wide dose range administered, we sought to retrospectively relate the efficacy of fulvestrant administered at a clinically relevant dose to that observed for more recently developed oral SERDs. To validate this comparison between studies, we first compared the average fold change in tumor volume for the vehicle control groups of these studies over 4 weeks of treatment and found them to be nearly identical (Online Resource 2a). As shown in Fig. 2a, oral SERDs AZD9496, GDC0810, and bazedoxifene (BZA) inhibited the growth of TamR xenograft tumors with similar efficacy. As observed for the fulvestrant-treated TamR tumor study in Fig. 1, efficacy was not directly related to the extent of ER turnover; although all three SERDs significantly downregulated ER expression, a significant difference could be discerned between AZD9496 and GDC0810 with respect to ER turnover (Fig. 2b). This difference was not reflected in the efficacy with which these SERDs inhibited tumor growth.

Calculation of the percent inhibition of tumor growth over time (as compared to their respective vehicle groups) for both studies included in Figs. 1 and 2 demonstrated a very similar response to the clinically relevant (25 mg/kg) dose of fulvestrant and to the oral SERDs (Table 1).

**Table 1:** % Tumor growth inhibition of TamR tumors over 4 weeks of treatment

Days of treatment	Fulvestrant				AZD9496 (%)	GCD0810 (%)	BZA (%)
	25 (%)	50 (%)	100 (%)	200 (%)			
0	− 1	− 1	− 3	2	− 3	− 5	− 3
2	25	24	23	28	13	11	11
4	20	20	20	24	18	22	23
7	21	26	27	24	27	28	26
9	18	28	16	25	24	27	26
11	24	31	32	39	30	35	33
14	33	37	44	47	31	32	31
16	29	40	36	48	32	42	38
18	39	44	50	53	36	51	42
21	44	51	46	61	43	49	48
23	45	58	53	66	47	53	48
25	47	59	49	67	46	53	46
28	47	62	58	74	43	52	41

### Fulvestrant and oral SERDs exhibit similar efficacy in a xenograft model of resistance to estrogen deprivation

The tamoxifen-resistant tumor model used in both of the above studies has proven predictive of clinical response of patients having endocrine therapy-resistant breast cancer to SERD administration in the clinic [18, 19]. However, the current clinical standard for first-line endocrine therapy is administration of an aromatase inhibitor (AI), enzymatic inhibitors of aromatase that block conversion of androgens to estrogens. Recent findings have shown that *ESR1* mutations conferring constitutive activity upon ER likely underlie resistance to AI therapy in a subset of patients, and these may impact subsequent response to SERD therapies [20–22]. However, a majority of patients lack detectable mutations in ER upon progression during AI treatments. In order to model this subset of patients, we have developed a long-term estrogen deprived (LTED) tumor model. Briefly, estrogen treatment was withdrawn from a ovariectomized *Nu:J* mouse bearing an actively growing estrogen treated MCF7 xenograft tumor (Online Resource 3a). Following an approximate 6-months regression and stasis, the tumor regained exponential growth and became the founder tumor of an LTED xenograft tumor model that retained ER expression and can be transplanted between ovariectomized mice.

**Fig. 3:**
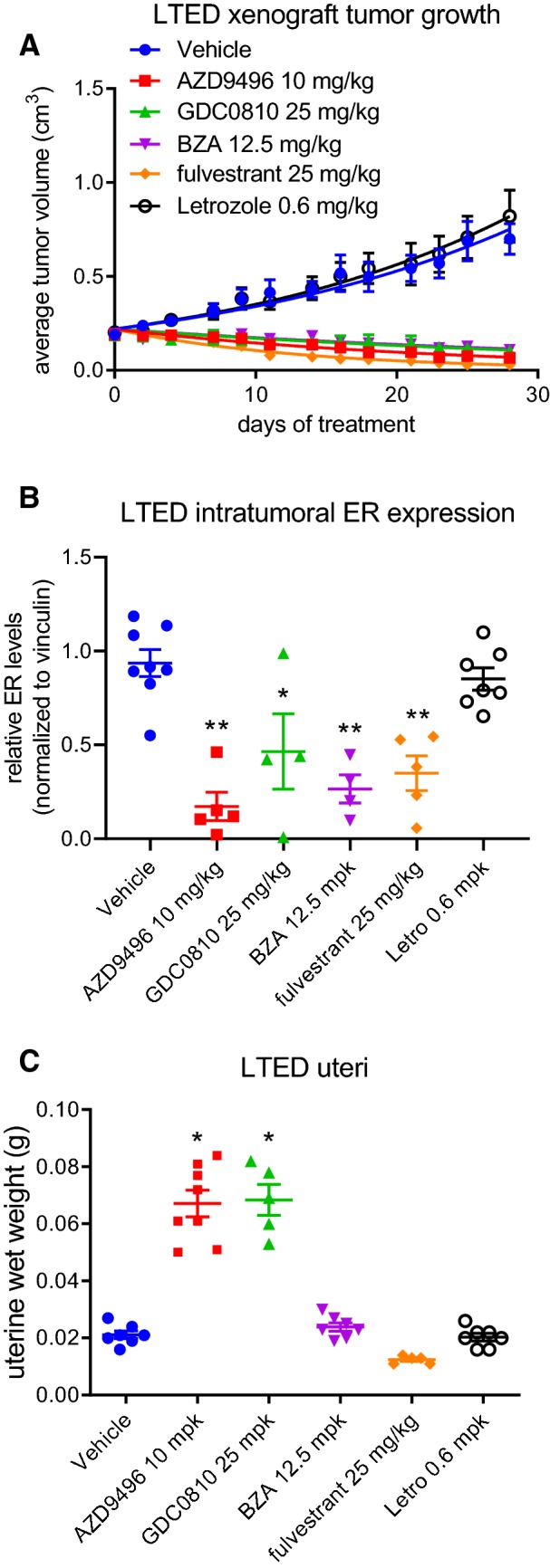
The clinically relevant doses of SERDs exhibited similar efficacy when compared using an estrogen deprived model of aromatase inhibitor resistance. **a** Ovariectomized *Nu:J* mice bearing LTED (long-term estrogen deprived) xenograft tumors were randomized to treatment with vehicle (po qd), AZD9496 (10 mg/kg po qd), GDC0810 (25 mg/kg po qd), BZA (12.5 mg/kg po qd) or fulvestrant (25 mg/kg sc qw). Data presented indicate the average tumor volume for each group (mean ± SEM) at each time point. *As compared to the vehicle control, significant (*p* > 0.05) inhibition of tumor growth was observed for all treatments, while no significant differences were noted between treatments (2-way ANOVA analysis followed by Bonferroni multiple comparison test). **b** ER levels present in tumors harvested from mice included in **a** were analyzed by western blotting of tissue extracts. A significant (*p* < 0.05) decrease in ER levels was observed for all SERD treatments (1-way ANOVA analysis followed by Bonferroni multiple comparison test). Primary western blot images are included in Online Resource Fig. 3b. **c** Weights of uteri excised after euthanasia were recorded as a measure of uterine stimulation. *A significant (*p* < 0.05) increase in uterine weight was observed for all AZD9496 and GDC0810 (1-way ANOVA analysis followed by Bonferroni multiple comparison test)

To compare the therapeutic and pharmacodynamic response of the LTED tumor model to SERD treatment, LTED tumors were established orthotopically in ovariectomized *Nu:J* mice. When tumors measured 0.1–0.15 cm^3^ volume, mice were randomized to 4 weeks of treatment with vehicle or clinically relevant doses of fulvestrant, AZD9496, GDC0810, BZA, or the AI letrozole. Letrozole was without effect on the growth of the LTED tumors, while all four SERDs similarly inhibited tumor growth (Fig. 3a). Comparison of the ER levels present in tumors harvested following the end of treatment demonstrated a similar extent of downregulation by all of the SERDs administered (Fig. 3b). These molecules fall largely into three chemical categories: steroidal SERDs (fulvestrant, RU58,668), acidic SERDs (GW5638 (etacstil), GDC0810, AZD9496), and basic SERDs (BZA, Rad1901 (elacestrant)). As these treatments represent different molecular subclasses of SERDs, we analyzed the effect of these regimens on uterine wet weight in the LTED tumor bearing mice following euthanasia. The rodent uterus has been used extensively to evaluate estrogenicity of compounds such as environmental estrogens and, as in this case, SERMs and SERDs. We observed no response of uterine weight to BZA or to letrozole, while a non-significant trend toward reduced uterine weight was observed for fulvestrant (Fig. 3c). Both AZD9496 and GDC0810 significantly increased uterine weight, which may suggest estrogen-like regulation of some aspects of ER activity by these compounds in this tissue.

**Fig. 4:**
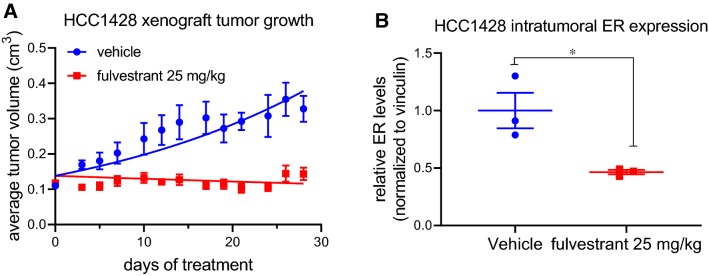
A clinically relevant dose of fulvestrant inhibited estrogen dependent tumor growth. **a** Ovariectomized *Nu:J* mice bearing HCC1428 breast cancer xenograft tumors were randomized to treatment with vehicle or fulvestrant (25 mg/kg sc qw). *As compared to the vehicle control, significant (*p* > 0.05) inhibition of tumor growth by fulvestrant was observed for multiple time points. **b** ER levels present in tumors harvested from mice included in **a** were analyzed by western blotting of tissue extracts. A significant (*p* < 0.05) decrease in ER levels was observed for fulvestrant treatment. Primary western blot images are included in Online Resource Fig. 3c

Both of the above xenograft models originated with the exquisitely estrogen dependent MCF7 breast cancer cell line, and these models were further adapted to ER-dependent growth without estrogen treatment. Therefore, an additional breast cancer cell model was utilized to determine the broader applicability of the administration of a clinically relevant dose of fulvestrant. HCC1428 breast cancer xenograft tumors were implanted orthotopically in ovariectomized *Nu:J* mice receiving oral estradiol (0.75 µg/kg) treatment, a dose insufficient to support MCF7 tumor growth and intended to model those patients exhibiting incomplete estradiol suppression during AI therapy. Upon reaching 0.1–0.13 cm^3^ volume, mice were randomized to 4 weeks of vehicle or fulvestrant (25 mg/kg) treatment. Tumor stasis in the treated animals was observed in this xenograft model as well, showing that a clinically relevant dose of fulvestrant adequately opposed the stimulatory effects of estrogen in this model with a modest but significant downregulation of ER expression (Fig. 4).

## Discussion

The initial preclinical reports of the activity of fulvestrant (ICI 182,780) described a compound with promise as a mechanistically novel approach to targeting ER activity in breast cancer, and indeed, in most preclinical models fulvestrant exhibited efficacy under conditions in which other contemporary drugs (e.g., tamoxifen, raloxifene, toremifene) were ineffective. It is still largely regarded as the only ER modulator that exhibits complete antagonist activity in vivo in all estrogen responsive tissues analyzed thus far. The initial clinical results, however, were far less promising; in the EFECT trial that enrolled patients that had already progressed during AI treatment, fulvestrant (250 mg monthly) exhibited efficacy comparable to that observed for the steroidal AI anastrozole, with a 7.4% response rate and nearly identical time to progression [23]. These results largely led to general conclusion within the field that upon resistance to one endocrine therapy, breast cancer patients were unlikely to benefit from further targeting of the ER pathway, and interest in further SERD development rapidly waned.

Two key observations renewed interest in SERD development and enabled development of additional SERD compounds. First, pharmacokinetic analyses of plasma exposure at increasing doses of fulvestrant indicated that a higher dose (500 vs. 250 mg/month), and a loading regimen in the first month of exposure (500 mg on d1, d14, d29) resulted in an improved response rate and increased time to progression [24–27]. Indeed, positron emission tomography/computed tomography (PET/CT) revealed that 30% of patients exhibited intratumoral [(18)F]fluoroestradiol (FES) binding during follow-up PET scans despite months of fulvestrant treatment using the originally approved (250 mg/month) treatment regimen to downregulate ER, evidence of incomplete saturation of ER binding, and insufficient fulvestrant exposure to sufficiently downregulate ER expression and/or antagonize agonist binding [28]. However, treatment-associated mutations of ER that reduce the binding affinity of fulvestrant may also contribute to low saturation of the receptor [21, 22]. Further evaluation of this higher dose regimen in comparison to anastrozole in advanced breast cancer patients confirmed that increasing fulvestrant exposure yielded likewise increased time to progression and overall survival as compared to anastrozole [3, 29, 30]. Therefore, pharmacokinetic liabilities of fulvestrant, and not the futility of further targeting of ER, likely underlies the initially low rate and duration of response observed. Secondly, the identification of an orally bioavailable non-steroidal SERD (GW5638, Etacstil) that was effective in preclinical models of endocrine therapy-resistant breast cancer and exhibited efficacy in an investigator initiated clinical trial clearly indicated that additional compounds having SERD activity could be identified and developed for clinical use [19, 31–33].

The past decade has witnessed a dramatic increase in the number of SERDs in development and entering clinical trials [34–39, 40]. Throughout their preclinical development, these compounds have been compared to fulvestrant, both in vitro and in vivo, with the goal of achieving equal or better ER downregulation and inhibition of receptor signaling in vivo as compared to fulvestrant. Comparison of the efficacy of these newly discovered compounds to a therapeutically unachievable fulvestrant exposure level may result in the selection of compounds having potential clinical liabilities at the expense of the development of other SERD compounds that might prove more tolerable. Indeed, development of several of these compounds have been discontinued following Phase I or Phase II trials for reasons relating to intolerable side effects or unexpected lack of efficacy.

In light of the difficulties experienced in advancing recently developed SERDs to the clinic, it is important to note that in at least one tumor model equivalent inhibition of tumor growth was observed for all doses of fulvestrant administered, despite the fact that post-study analysis of the tumor tissues indicated that the clinically relevant dose (25 mg/kg) did not result in significant ER downregulation. These data are in agreement with our prior in vitro findings demonstrating that the antagonist activity of fulvestrant is sufficient to block ER activity, while receptor turnover is dispensable [41]. These data raise the question whether the current path(s) of SERD development place unnecessary emphasis on potent and efficient downregulation of the receptor without equal attention to the efficacy of ER inhibition independent of receptor degradation. Furthermore, a recent study has reported that the efficacy of fulvestrant and additional SERDs are mediated by their ability to slow receptor mobility as opposed to their ability to downregulate ER [42]. In essence, the imperative to develop a SERD may result in the unnecessary omission of potentially tolerable complete antagonists and/or high affinity SERMs. We believe that drug exposure and target engagement are likely to be that which has limited SERM and SERD efficacy evaluated thus far, as opposed to incomplete ER downregulation.

It is surprising that a thorough pharmacokinetic evaluation of fulvestrant in preclinical models had not been completed prior to the current study. A direct comparison of the 25 mg/kg dose predicted to be clinically relevant (based upon body surface area calculation) to that widely used in the field (5 mg/mouse; 200 mg/kg) indicates that the lower dose exhibits exposure comparable to that observed clinically and also efficacy in two in vivo models of endocrine therapy-resistant breast cancer. Therefore, it is anticipated that the results of this study will provide guidance for how to appropriately evaluate ER modulators and compare their efficacy to standard of care endocrine agents at exposures that are clinically relevant.

## Electronic supplementary material

Below is the link to the electronic supplementary material.

Supplementary material 1 (PDF 374 kb)
