# Correction to: Pharmacokinetic and pharmacodynamic analysis of fulvestrant in preclinical models of breast cancer to assess the importance of its estrogen receptor-α degrader activity in antitumor efficacy

**DOI:** 10.1007/s10549-019-05498-0

**Published:** 2019-11-16

**Authors:** Suzanne E. Wardell, Alexander P. Yllanes, Christina A. Chao, Yeeun Bae, Kaitlyn J. Andreano, Taylor K. Desautels, Kendall A. Heetderks, Jeremy T. Blitzer, John D. Norris, Donald P. McDonnell

**Affiliations:** 1 Department of Pharmacology and Cancer Biology, Duke University School of Medicine, Box 3813, Durham, NC 27710 USA; 2 Potrero Hill Therapeutics, San Francisco, CA 94110 USA

## Correction to: Breast Cancer Research and Treatment 10.1007/s10549-019-05454-y

The article Pharmacokinetic and pharmacodynamic analysis of fulvestrant in preclinical models of breast cancer to assess the importance of its estrogen receptor-α degrader activity in antitumor efficacy, written by Suzanne E. Wardell, Alexander P. Yllanes, Christina A. Chao, Yeeun Bae, Kaitlyn J. Andreano, Taylor K. Desautels, Kendall A. Heetderks, Jeremy T. Blitzer, John D. Norris, Donald P. McDonnell, was originally published electronically on the publisher’s internet portal on September 27, 2019 without open access.

With the author(s)’ decision to opt for Open Choice the copyright of the article changed on November 16, 2019 to © The Author(s) 2019 and the article is forthwith distributed under a Creative Commons Attribution 4.0 International License (https://creativecommons.org/licenses/by/4.0/), which permits use, sharing, adaptation, distribution and reproduction in any medium or format, as long as you give appropriate credit to the original author(s) and the source, provide a link to the Creative Commons licence, and indicate if changes were made. The original article has been corrected.

