# Supplementary material for: Pharmacokinetic and pharmacodynamic analysis of fulvestrant in preclinical models of breast cancer to assess the importance of its estrogen receptor-α degrader activity in antitumor efficacy
Source: Breast Cancer Res Treat. 2019 Sep 27;179(1):67–77. doi: 10.1007/s10549-019-05454-y (PMC6985185; doi:10.1007/s10549-019-05454-y)
Supplement: Supplementary file 1 — Supplementary material 1 (PDF 374 kb) [file 10549_2019_5454_MOESM1_ESM.pdf]

Online Resource 1

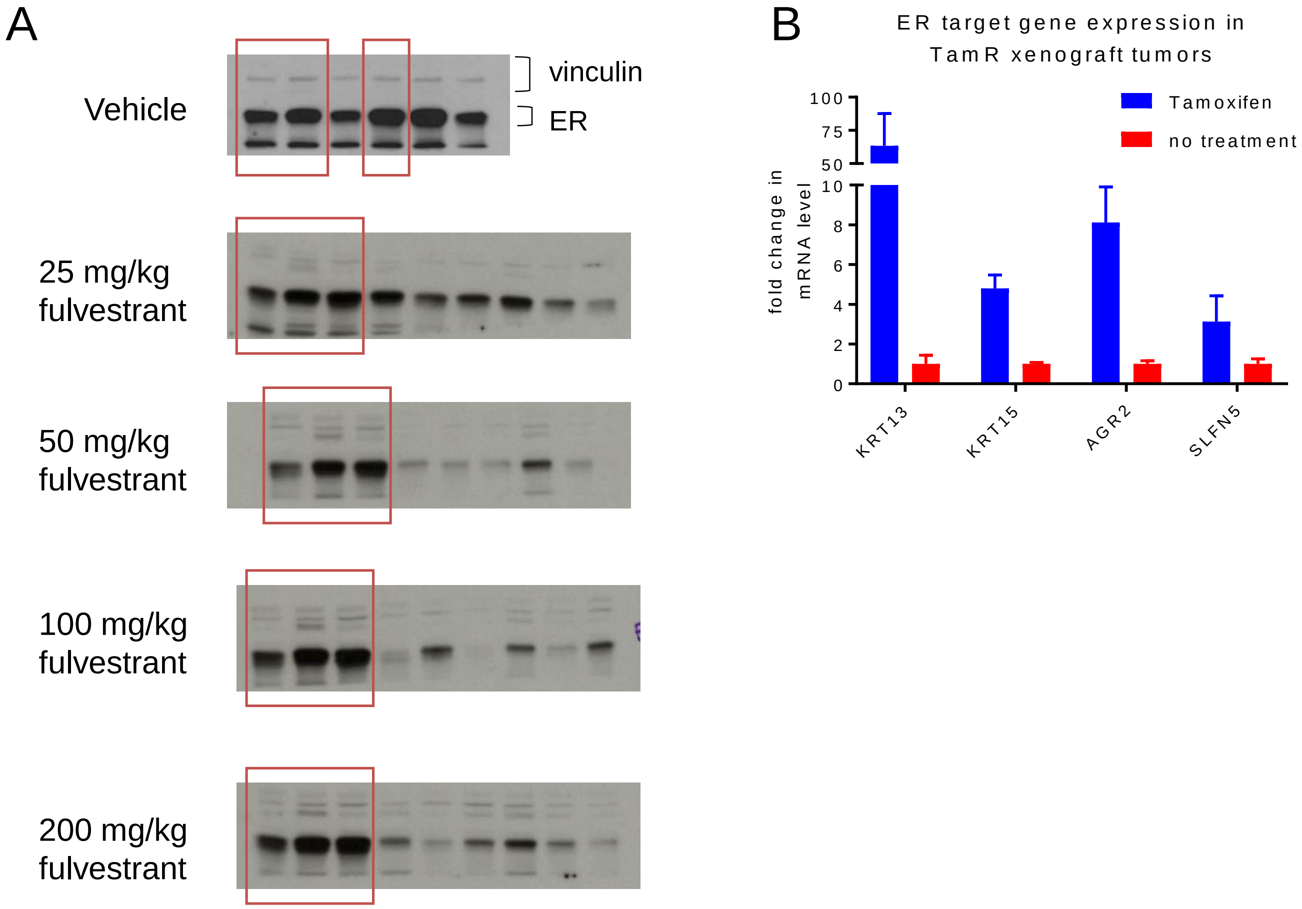

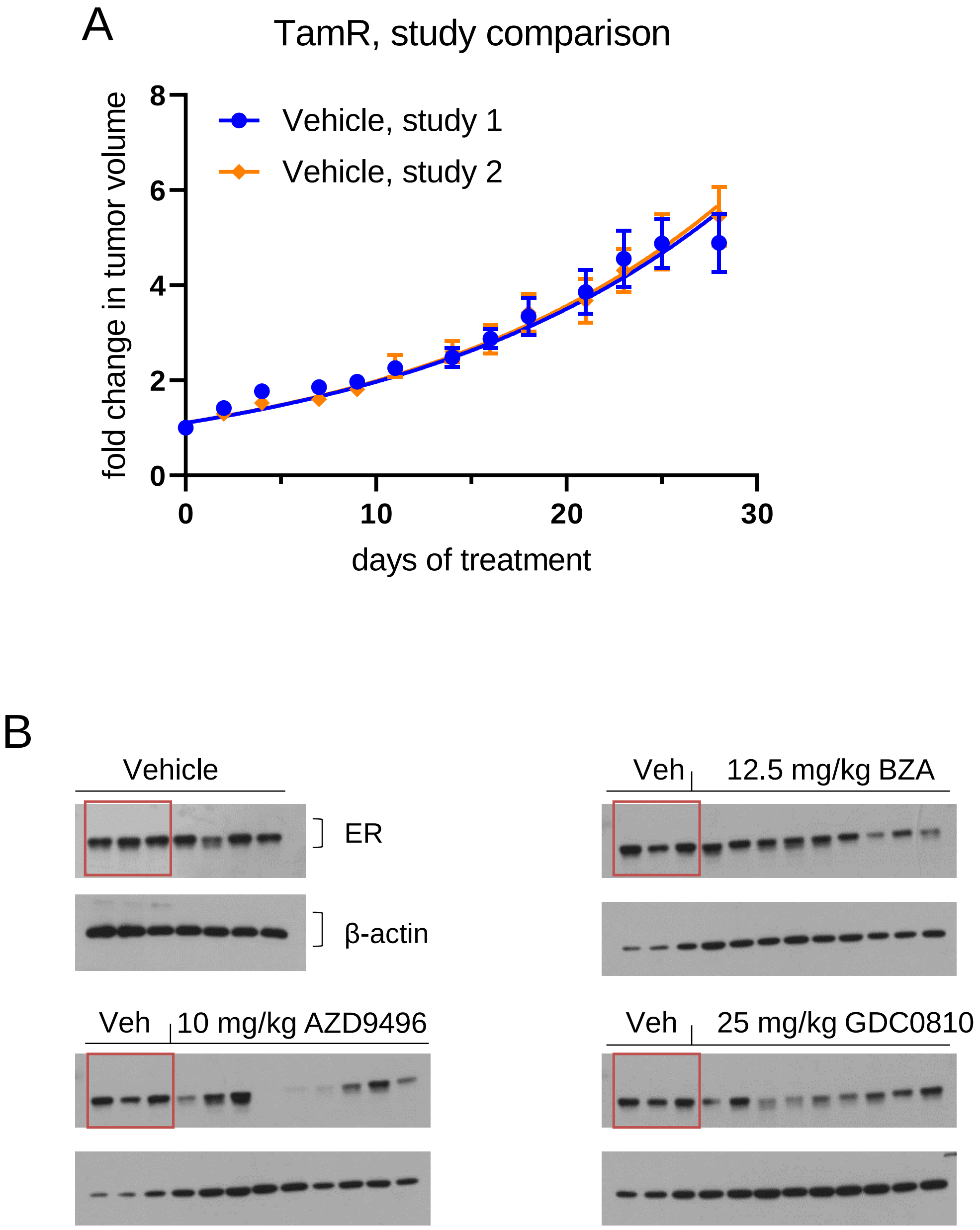
A
TamR, study comparison
Vehicle, study 1
Vehicle, study 2
fold change in tumor volume
0
2
4
6
8
0
10
20
30
days of treatment
B
Vehicle
ER
β-actin
Veh
12.5 mg/kg BZA
Veh
10 mg/kg AZD9496
Veh
25 mg/kg GDC0810

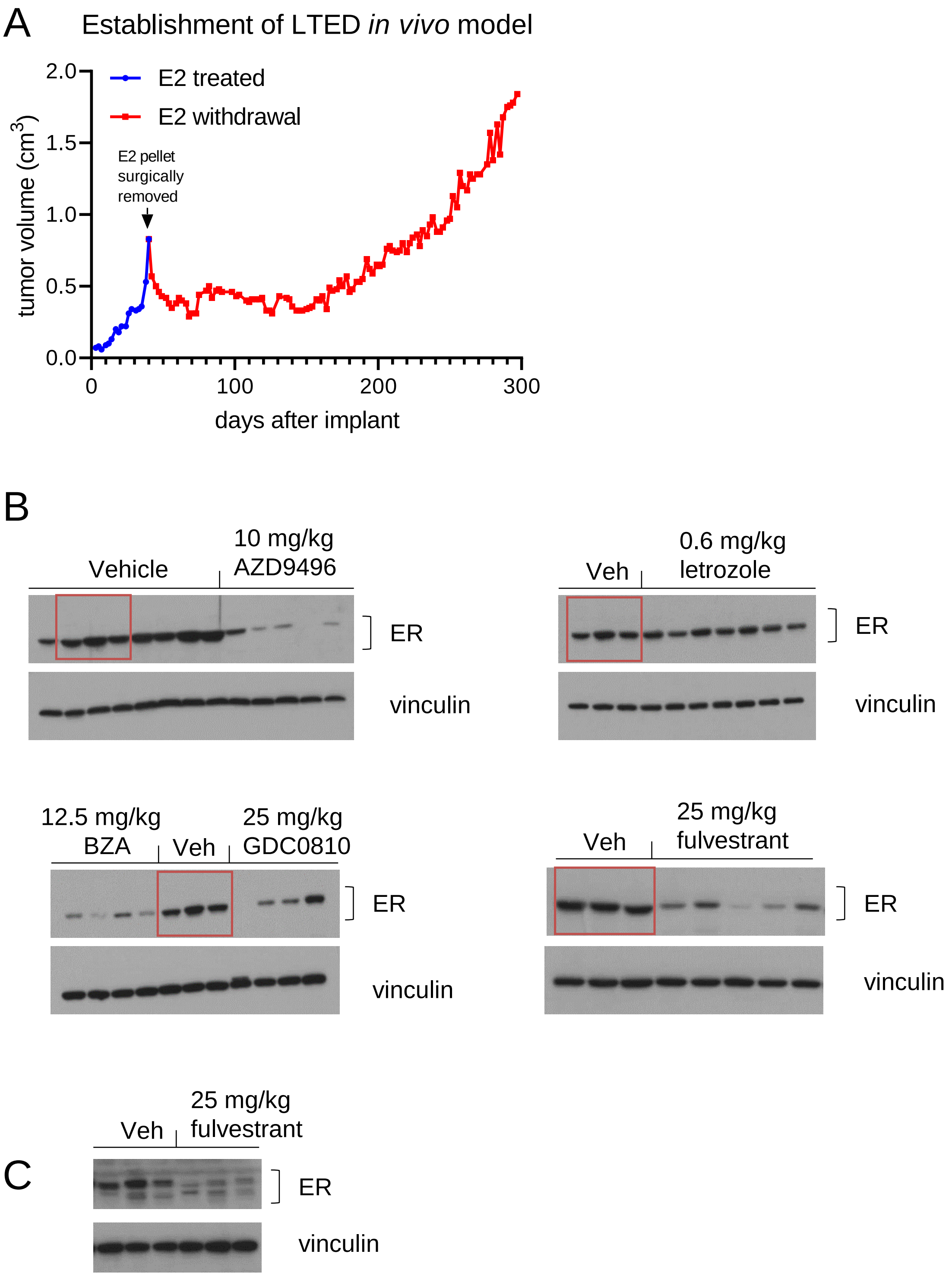
A
Establishment of LTED *in vivo* model
E2 treated
E2 withdrawal
E2 pellet
surgically
removed
tumor volume (cm^3)
2.0
1.5
1.0
0.5
0.0
0
100
200
300
days after implant
B
Vehicle
10 mg/kg
AZD9496
ER
vinculin
Veh
0.6 mg/kg
letrozole
ER
vinculin
12.5 mg/kg
BZA
Veh
25 mg/kg
GDC0810
ER
vinculin
Veh
25 mg/kg
fulvestrant
ER
vinculin
C
Veh
25 mg/kg
fulvestrant
ER
vinculin

Wardell et al, captions for online resources

Online Resource 1. A) Tamoxifen treated *Nu:J* mice bearing TamR (tamoxifen resistant) xenograft tumors were randomized to treatment with vehicle or fulvestrant (25 – 200 mg/kg). ER levels present in tumors harvested from mice were analyzed by western blotting of tissue extracts to detect ER and vinculin. Indicated by red boxes are tumor extracts from the vehicle group that were included on all blots to enable normalization between blots. ER levels (normalized to vinculin) in treated tumors were calculated relative to the average normalized ER levels detected in the vehicle samples included in the same blot. B) RNA was isolated TamR tumors implanted in mice receiving tamoxifen treatment (5 mg/60 day implanted pellet) or no treatment. Expression of ER target genes, relative to housekeeping gene 36B4, was detected by real time quantitative PCR.

Online Resource 2. A) Average fold change in tumor relative to the initial volume at randomization is shown for the vehicle treatment groups only for both TamR xenograft studies presented in Figures 1 and 2. No significant difference was detected between these growth curvest (2-way ANOVA followed by Bonferroni multiple comparison test). B) Intratumoral ER levels present in the TamR tumors harvested from mice enrolled in the treatment study presented in Figure 2 were analyzed by western blotting of tissue extracts to detect ER and vinculin. Indicated by red boxes are tumor extracts from the vehicle group that were included on all blots to enable normalization between blots. ER levels (normalized to β-actin) in treated tumors were calculated relative to the average normalized ER levels detected in the vehicle samples included in the same blot.

Online Resource 3. A) The LTED xenograft tumor model of resistance to estrogen deprivation was generated *in vivo* as illustrated. Estrogen treatment (0.72 mg/60 day implanted pellet) was withdrawn from an ovariectomized *Nu:J* mouse bearing an MCF7 xenograft tumor. After an initial 50% regression and approximate 3 months' stasis, the tumor resumed growth in an estrogen independent manner. These tumors can be subdivided and exhibit similar growth in ovariectomized and intact mice. B) Intratumoral ER levels present in the LTED tumors harvested from mice enrolled in the treatment study presented in Figure 3 were analyzed by western blotting of tissue extracts to detect ER and vinculin. Indicated by red boxes are tumor extracts from the vehicle group that were included on all blots to enable normalization between blots. ER levels (normalized to vinculin) in treated tumors were calculated relative to the average normalized ER levels detected in the vehicle samples included in the same blot. C) Intratumoral ER levels present in the HCC1428 tumors harvested from mice enrolled in the treatment study presented in Figure 4 were analyzed by western blotting of tissue extracts as described in (B).
